# A Doctor’s Habits

**Published:** 1859-08

**Authors:** 


### A DOCTOR'S HABITS.

Sydenham, one of the most eminent physicians within three hundred years, says that on rising, he drank a cup or two of tea, then rode in his carriage until noon; took meat in great moderation for dinner, and wine after, then rode in his coach; ate no supper, but drank beer instead, and in getting into bed drank wine. He died at the comparatively early age of sixty-five, having suffered from the gout for a quarter of a century.

Sydenham practised medicine from observation, rather than books, and by his success, became the most eminent physician in England; and yet, he was not observant enough as to himself, to keep him from being a confirmed invalid, nor wise enough to know, that breakfasting on tea, and supping on beer, and bedding on wine, (and these alone) with a plentiful supply of the latter for dinner also, was quite enough to give him, or any body else, the gout, and curtail human life. Had he lived on plain substantial food, eating twice a day, and spent the hours of forenoon and afternoon on horseback, instead of riding at his ease in a carriage, he would, with great probability, have lived in good health to the age of eighty years, without the tea, and wine and beer.

